# H_2_O_2_ Induces Calcium and ERMES Complex-Dependent Mitochondrial Constriction and Division as Well as Mitochondrial Outer Membrane Remodeling in *Aspergillus nidulans*

**DOI:** 10.3390/jof8080829

**Published:** 2022-08-09

**Authors:** Verónica Garrido-Bazán, Jesús Aguirre

**Affiliations:** Departamento de Biología Celular y del Desarrollo, Instituto de Fisiología Celular, Universidad Nacional Autónoma de México, Apartado Postal 70-242, Ciudad de México 04510, Mexico

**Keywords:** ROS signaling, mitochondrial dynamics, mitochondrial fission, endoplasmic reticulum, Drp1, ERMES, calcium

## Abstract

The dynamin-like protein DnmA and its receptor FisA are essential for H_2_O_2_-induced mitochondrial division in *Aspergillus nidulans*. Here, we show that in the absence of DnmA or FisA, mitochondria show few spontaneous transient constrictions, the frequency of which is extensively increased by H_2_O_2_ or the carbonyl cyanide m-chlorophenyl hydrazone (CCCP). While H_2_O_2_-induced constrictions are transient, CCCP induces a drastic and irreversible alteration of mitochondrial filaments. H_2_O_2_ induces a gradual mitochondrial depolarization, while CCCP-induced depolarization is abrupt. The calcium chelator BAPTA-AM prevents the formation of mitochondrial constrictions induced by either H_2_O_2_ or CCCP. H_2_O_2_ also induces major rearrangements of the mitochondrial outer membrane, which remain after constrictions dissipate, as well as changes in endoplasmic reticulum (ER) and nuclear morphology. Similar mitochondrial constriction, ER and nuclear morphology changes are detected during the early stages of asexual development. ER and ER-Mitochondria encounter structure (ERMES) complex—composed of proteins Mdm10, Mmm1, Mdm43 and Mdm12—are important for mitochondrial division in *Saccharomyces cerevisiae*. As the Mdm10 ortholog MdmB was found to be essential in *A. nidulans*, we evaluated its functions in *ΔmdmB* terminal mutants and *ΔmdmB* heterokaryons. *ΔmdmB* conidia produce a short germ tube that fails to grow further, in which inherited mitochondria become gigantic and round shaped, lacking clear contacts with the ER. In slow-growing *ΔmdmB* heterokaryotic mycelia, multiple hyphae contain very long mitochondria with high ROS levels, as occur in *ΔdnmA* and *ΔfisA* mutants. In this hyphae, H_2_O_2_ fails to induce mitochondrial constrictions but not outer mitochondrial membrane reshaping, indicating that these are two separate effects of H_2_O_2_. Our results indicate that H_2_O_2_ induces a generalized mitochondrial constriction response, prior to actual division, involving gradual depolarization; they also indicate that Ca^2+^ and the ERMES complex are critical for both mitochondrial constriction and division. This supports a view of mitochondrial dynamics as the result of a cascade of signaling events that can be initiated in vivo by H_2_O_2_.

## 1. Introduction

We are interested in understanding the roles of reactive oxygen species (ROS) as physiological regulatory signals [1,2]. ROS play critical roles in cell differentiation in fungi, where ROS-producing NADPH oxidases are essential for sexual differentiation [3,4,5,6,7]. Consistent with this, mutants affected in components of the antioxidant response show premature sexual differentiation and decreased asexual development [8,9].

H_2_O_2_ is considered to be the most relevant ROS in redox biology [10]. Indeed, in the fungus *Aspergillus nidulans*, H_2_O_2_ activates the MAPKs SakA, MpkC [8,11,12], MpkA and MpkB [13], and changes the phosphorylation patterns of multiple other proteins involved in signal transduction, gene expression, metabolism and development [13]. SakA mediates the nuclear localization of its substrate SrkA, and when SakA is not present, H_2_O_2_ induces SrkA mitochondrial localization [14]. Under these conditions, H_2_O_2_ also induces a massive mitochondrial division [14].

Mitochondria are major sources of ROS, and their replication and maintenance depend on the fission, fusion and segregation of pre-existing organelles. Mitochondria change their morphology throughout the cell cycle, and actively divide to allow proper segregation to daughter cells during mitosis [15]. Mitochondrial division, also a mechanism to segregate damaged parts for their posterior degradation by mitophagy [16], is coordinated by membrane-associated proteins, influenced by interactions with the actin cytoskeleton and other organelles [17] such as the endoplasmic reticulum (ER), and executed by dynamin-related proteins (Drp1 in humans and other animals; DnmA in *A. nidulans*). These GTPases, which are highly conserved in all eukaryotes, oligomerize in ring-like structures around the mitochondrial tubules, where GTP hydrolysis induces a conformational change that allows mitochondrial division [18,19,20]. Mutations in Drp1 and DnmA cause embryonic lethality in mice [21] and drastic defects in growth and development in *A. nidulans* [22], respectively. Moreover, defects in mitochondrial division are associated to different human pathologies [23]. In *Saccharomyces cerevisiae* and other fungi, Dnm1 is recruited to mitochondria by the adaptor protein Fis1 (FisA in *A. nidulans*) together with adaptors Mdv1 and Caf4 [24,25,26]. In *A. nidulans*, H_2_O_2_-induced mitochondrial division is totally dependent on DnmA and its receptor FisA [22].

*A. nidulans* is an excellent model to study cell biology processes during growth and cell differentiation, as it undergoes both asexual and sexual development. Vegetative hyphae are highly polarized cells that undergo branching and grow at the tips. Asexual development (conidiation) involves the formation of complex conidiophore structures. During this process, conidiophore stalk cells grow for a fixed length towards the air interface and then develop a multinucleated vesicle, from which uninucleate cells called metulae emerge. Metulae differentiate into conidiogenic cells called phialides, which produce long chains of uninucleate spores (conidia) by a series of mitotic divisions [27]. Actively growing hyphae from mutants lacking DnmA or FisA produce very long, tubular mitochondria, which fail to undergo division. Nevertheless, at the vesicle stage, mitochondria form highly convoluted structures that branch through developing metulae and phialides in a process that results in the partitioning of a single mitochondrion to every developed conidia [22].

Here, we show that even in the absence of DnmA or FisA, H_2_O_2_ induces major changes in mitochondrial morphology, characterized by an extensive production of mitochondrial constrictions that are preceded by mitochondrial depolarization. We show that calcium and ERMES complex subunit MdmB are required for mitochondrial constriction and division but not for depolarization or H_2_O_2_-induced changes in the mitochondrial outer membrane. In addition, H_2_O_2_ elicited changes in the endoplasmic reticulum (ER) and nuclear morphology that were similar to those observed during conidiophore development.

## 2. Materials and Methods

### 2.1. Strains, Media, and Growth Conditions

The *Aspergillus nidulans* strains used in this work are listed in Table S1. All of the strains were grown at 37 °C in 1% glucose–nitrate solid minimal medium [28], plus supplements.

### 2.2. Gene Deletion and Organelle Labelling

In order to delete the *mdmB* gene (AN6901), a construction was made using double-joint PCR [29], different combinations of primers (Table S2), and genomic DNA as a template. Two PCR fragments were generated using primers 5′ForMdmB/5′RevMdmB and 3′ForMdmB/3′RevMdmB. The *Aspergillus fumigatus pyrG* gene, used as selective marker, was amplified from plasmid PFNO3 [30] with primers PyrGForward and PyrGReverse. These three fragments were purified, mixed and used in a fusion PCR with primers 5’NestMdmB and 3′NestMdmB. The final 3856 bp mdmB–AfpyrG–mdmB cassette was purified and used to transform *A. nidulans* strains CDV02 and CVG29 by electroporation [31,32]. Several heterokaryons were obtained in three different transformations, three of which were analyzed by PCR and maintained on selective medium.

In order to label the endoplasmic reticulum, the wild-type strain (CLK43) was transformed with plasmid pAM01 [33], which contains ER-signal and ER-retention sequences from *Podospora anserina* BiP chaperone protein fused to GFP, expressed from *A. nidulans gpdA* promotor. The presence of labelled ER was confirmed by epifluorescence microscopy in strain TVG6. Strains TVG6 and CVG8 (with labelled mitochondria) were crossed to obtain strain CVG34, containing ER labelled with GFP and mitochondria labelled with mCherry. Strains CVG34 and TVG1(*ΔdnmA*) were crossed to obtain CVG32 (*ΔdnmA* with labelled ER and mitochondria). Strains CVG32 and CVG27 were crossed to obtain strains CVG46 (WT) and CVG47 (*ΔdnmA*) with labelled ER, mitochondria and nuclei. In order to obtain strains with labelled mitochondrial outer membranes, strain CDV1, which expresses Tom20 [34] homolog AN0559 fused to GFP, from the *A. nidulans gpdA* promoter [35], was crossed with strain CVG1. From this cross, strains CVG45 (*ΔdnmA* with matrix and outer-membrane labelled mitochondria) and CVG48 (*ΔdnmA* with labelled mitochondrial outer membrane) were obtained. In all cases, the *dnmA* deletion for was confirmed by PCR.

### 2.3. Confocal and Airyscan Microscopy

The images were captured using a Zeiss LSM800 inverted laser scanning confocal microscope (Carl Zeiss, Jena, Germany) using a Plan Apochromat 63×/1.4 oil immersion objective and 405, 488 and 561 laser lines, according to the fluorescent tag. The Airyscan images were obtained with a LSM800 microscope and a Plan-Apochromat 63×/1.4 oil DIC M27 objective, using the Airyscan detectors (Carl Zeiss, Jena, Germany), with a 32-channel array of GaAsP detectors configured at 1.25 airy units per channel. Airyscan microscopy uses a confocal detection system that more efficiently collects light, increasing the signal-to-noise ratio and image speed acquisition, which results in higher resolution imaging than conventional confocal microscopy. All of the observations were conducted at 35 °C. The images were processed using software Zen 2012 (Carl Zeiss, Jena, Germany) and ImageJ 2 (v2.030/1.53f, K. W. Elicerei, Madison, USA). For fixed images, the samples were incubated with 300 µL of a 1:100 dilution of stock fixing solution (2.46 M paraformaldehyde, 0.44 M EGTA, 0.5 M MgCl, 100 mM PIPES pH 6.9) for 3 min, rinsed three times with water, and observed using epifluorescence or confocal microscopy. TMRM (tetramethylrhodamine, methyl ester) was used to detect mitochondrial membrane potential [36]. A 10 mM stock solution prepared in DMSO was maintained frozen. Then, 100 µM working solutions were prepared with water and used to cover sections of solid medium containing growing mycelia, for 20 min at 37 °C. The TMRM solution was removed and mycelia was treated or not with 5 mM H_2_O_2_, and observed using confocal microscopy. A 1 mM CCCP (Carbonyl cyanide m-chlorophenyl hydrazone, Sigma Aldrich, St. Louis, USA) stock solution was prepared in ethanol and maintained frozen. A 10 µM working solution was prepared with water and used to cover sections of solid medium containing growing mycelia for 20 min. After this, the samples were rinsed three times with water and observed using confocal microscopy. Permeant calcium chelator BAPTA-AM (Invitrogen, Waltham, MA, USA) was prepared in DMSO as a 15 mM stock solution, and was maintained frozen. Then, 200 µM working solutions prepared with water were used to cover mycelial samples for 2 h at 37 °C. After this, BAPTA-AM was removed, and the samples were rinsed with water and treated or not with 5 mM H_2_O_2_ for 20 min. Then, the samples were fixed or observed using confocal microscopy. All of the hyphal observations were restricted to growing tips, in order to avoid changes in mitochondrial morphology due to senescence or starvation. For the conidiophore imaging, the growing edge of a colony grown for 3 days at 37 °C was sectioned, a drop of water was added, and the section was carefully covered with a coverslip. Fields with early development conidiophores and a well-preserved structure were chosen for observation using confocal microscopy.

## 3. Results

### 3.1. H_2_O_2_ and CCCP Induce Mitochondrial Constriction and Membrane Depolarization 

**Figure 1:**
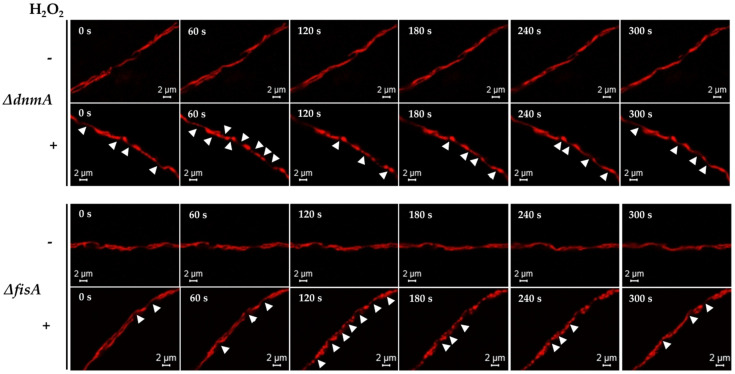
H_2_O_2_ induces transient mitochondrial constrictions in the absence of the mitochondrial fission machinery. Mycelia from *ΔdnmA* (CVG1) and *ΔfisA* (CVG2) mutant strains grown for 18 h were treated or not with 5 mM H_2_O_2_ for 20 min. After this, H_2_O_2_ was removed; after another 10-min incubation, hyphae were observed at the indicated times using confocal microscopy. The mitochondrial matrix is labelled with mts::mCherry. The arrowheads point to some of the constriction events.

We have shown that non-lethal concentrations of H_2_O_2_ induce widespread mitochondrial division in *A. nidulans* wild-type strains, and that proteins DnmA and FisA are necessary for this process [22]. The results in Figure S1 confirm that H_2_O_2_ induces the production of individual round mitochondria, which do not recover the filamentous form within the experimental time frame or even longer incubation times. We detected that in the absence of DnmA or FisA, H_2_O_2_ was still able to induce changes in mitochondrial morphology. In order to study these changes, we initially used 18-h grown mycelia from *ΔdnmA* and *ΔfisA* mutants with mCherry matrix labelled mitochondria. These mutants were treated or not with 5 mM H_2_O_2_ for 20 min. Then, H_2_O_2_ was removed and the mycelia were incubated for another 10 min. After this, the mycelia were immediately fixed and multiple hyphal tips were observed using confocal microscopy. In the absence of H_2_O_2_, the mitochondria from both *ΔdnmA* and *ΔfisA* mutants maintained a regular, filamentous form in 84–86% of the hyphal tips observed. However, a few isolated constriction-like structures were observed in 14–16% of the hyphal tips. In sharp contrast, in H_2_O_2_-treated samples, 81 and 79% of the hyphal tips from *ΔdnmA* and *ΔfisA* mutants contained mitochondria with multiple constriction-like structures, respectively (Figure S2). These results suggested that without H_2_O_2_, the mitochondria suffered some spontaneous and transient constrictions, and that the frequency of these constrictions was drastically increased by H_2_O_2_. In order to test this, we followed this process in vivo using the same experimental conditions. Indeed, without H_2_O_2,_ most *ΔdnmA* mitochondria maintained a filamentous form during the observation period (0–300 s). In contrast, H_2_O_2_ induced the formation of round structures and mitochondrial constriction-like structures between 0 and 180 s, which became less defined between 240 and 300 s when the filamentous form started to recover (Figure 1, top panels). Similar phenomena were observed in the *ΔfisA* mutant, although mitochondrial constriction-like structures were more evident between 120 and 240 s, and the filamentous morphology started to recover around 300 s (Figure 1, bottom panels).

**Figure 2:**
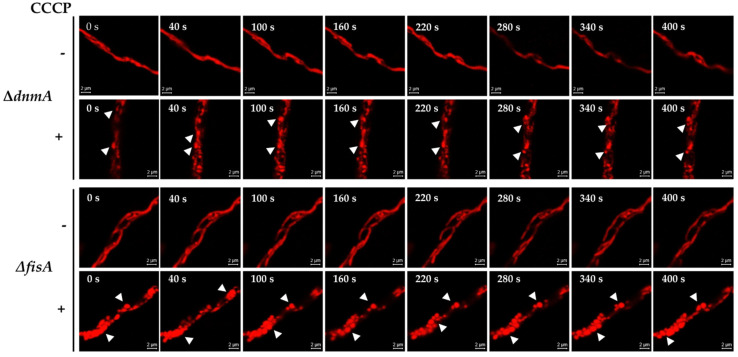
Uncoupler CCCP induces irreversible mitochondrial constrictions in the absence of the mitochondrial fission machinery. Mycelia from *ΔdnmA* (CVG1) and *ΔfisA* (CVG2) mutant strains grown for 18 h were treated or not with 10 μM CCCP for 20 min. After this, CCCP was removed, and the hyphae were incubated for another 10 min and observed at the indicated times using confocal microscopy. The mitochondrial matrix is labelled with mts::mCherry. The arrowheads point to some of the round structures produced by mitochondrial constrictions.

**Figure 3:**
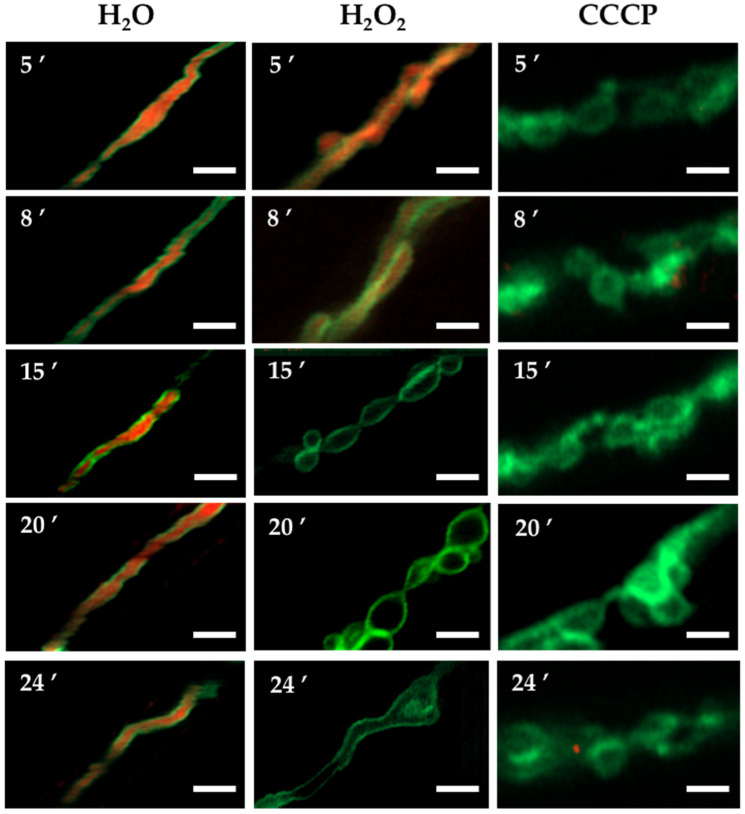
H_2_O_2_ induces a gradual depolarization of the mitochondria before extensive mitochondrial constrictions are observed. Mycelia from *ΔdnmA tom20::gfp* strain CVG48 were grown for 18 h at 37 °C and stained with 100 μM TMRM for 20 min. After this, samples were rinsed three times with distilled water and then treated or not with 5 mM H_2_O_2_ or 10 μM CCCP for another 20 min, rinsed, and immediately observed using Airyscan microscopy. The red florescence signal corresponds to TMRM-stained mitochondria, and the green signal corresponds to GFP-labelled mitochondrial outer membrane. Scale bar = 2 μm.

**Figure 4:**
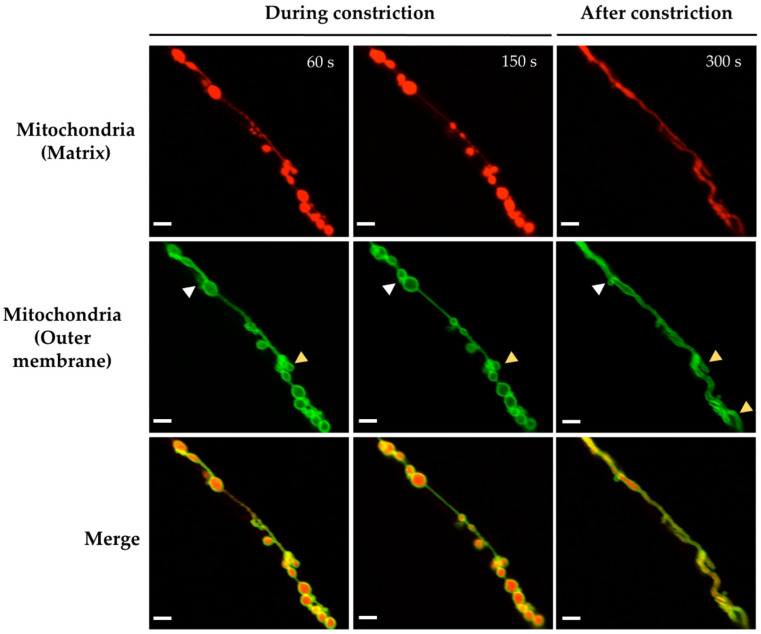
H_2_O_2_ induces mitochondrial branching and the major reshaping of the outer mitochondrial membrane. Mycelia from *ΔdnmA* (CVG45) mutant strain were grown for 18 h and then treated with 5 mM H_2_O_2_ for 20 min. H_2_O_2_ was removed, and after another 10 min of incubation, hyphae were observed at the indicated times using Airyscan microscopy. The white and yellow arrowheads point to septum-like and branching structures, respectively. Scale bar = 2 μm.

In order to explore whether the H_2_O_2_-induced constriction-like structures were related to mitochondrial membrane depolarization, we first exposed a wild-type strain to carbonyl cyanide *m*-chlorophenylhydrazone (CCCP), a well-known oxidative phosphorylation uncoupler [37]. Like H_2_O_2_, CCCP was able to induce a massive mitochondrial division in a wild-type background, and the mitochondria did not recover their filamentous form after prolonged incubation times (Figure S1). When *ΔdnmA* and *ΔfisA* mutants were treated with CCCP, it induced mitochondrial constriction-like structures in 87 and 89% of the hyphal tips observed in both *ΔdnmA* and *ΔfisA* mutants, respectively (Figure S2). Observations in vivo showed that CCCP induced constriction-like structures much faster than H_2_O_2_, and that the changes in mitochondrial morphology were more dramatic in the *ΔfisA* mutant (Figure 2). Notably, these CCCP-induced changes were not reversible, and the round structures remained up to 400 s and beyond. Because H_2_O_2_ induces similar responses in *ΔdnmA* and *ΔfisA* mutants, we decided to focus our analysis using only *ΔdnmA* mutants. In order to follow mitochondrial depolarization more directly, we incubated *ΔdnmA* mutant cells—in which the outer mitochondrial membrane was labelled with GFP—in the presence of the voltage sensor tetramethylrhodamine methyl-ester (TMRM), which is rapidly sequestered by the negatively charged mitochondrial membrane potential. The results in Figure 3 and Figure S3 show that after this incubation, about 95% of the hyphae contained TMRM-stained mitochondria. Under these conditions, H_2_O_2_ induced a gradual loss of mitochondrial membrane potential. After 5, 8, 15 and 20 min in H_2_O_2_, the number of hyphae stained with TMRM decreased to 87, 76, 20 and 6%, respectively. In contrast, in the presence of CCCP, mitochondrial membrane potential was lost after only 5 min of incubation (Figure 3). The use of strains with GFP-labelled outer mitochondrial membrane (Figure 3, Figure 4, Figure S4 and Figure S5) allowed us to confirm that the mitochondrial constriction-like structures observed in Figure 1 and Figure 2 correspond to actual constrictions present in non-dividing mitochondria; they will be named as such. Notably, H_2_O_2_-induced mitochondrial constrictions were evident at around 15 min, while with CCCP they were already present after 5 min (Figure 3).

We used the *ΔdnmA* strain with labelled matrix and outer mitochondrial membrane to follow the changes in both compartments induced by H_2_O_2_. The results in Figure 4 show that H_2_O_2_ induced both transient and more permanent changes in mitochondrial morphology within a short time frame. Indeed, the filamentous mitochondria went from a series of globular structures connected by thin filaments of external membrane, back to a filamentous form. In contrast, after most of the constrictions disappeared, the mitochondrial outer membrane presented major rearrangements, including the formation of mitochondrial branching points and septum-like structures (Figure 4 and Figure S4), as well as donut-like structures (Figure S4). Figure 4 also shows that although most constrictions produce globular shapes of similar size, some smaller round shapes were also produced. CCCP, in addition to irreversible mitochondrial constrictions, also induced the major reshaping of the mitochondrial outer membrane (Figure S5).

Our results indicate that H_2_O_2_ induces a generalized and transient mitochondrial constriction response prior to actual division, which involves gradual membrane depolarization, as well as major rearrangements of the outer mitochondrial membrane, some of which appear to be related to mitochondrial branching.

### 3.2. H_2_O_2_-Induced Mitochondrial Constriction Sites Are in Contact with the Endoplasmic Reticulum

**Figure 5:**
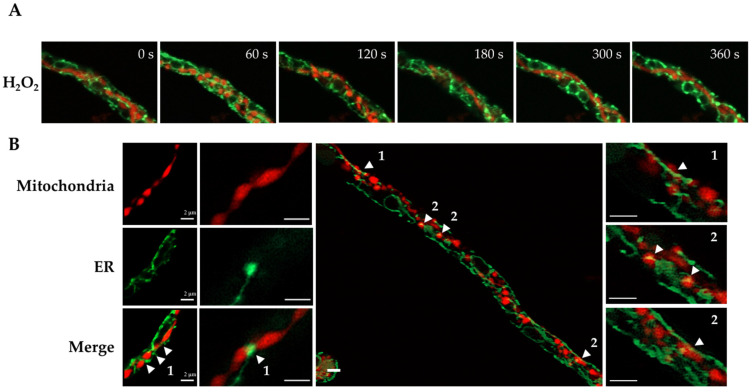
H_2_O_2_-induced mitochondrial constriction sites are in close contact with the endoplasmic reticulum. Mycelia from *ΔdnmA* (CVG32) mutant strain was grown for 18 h and treated with 5 mM H_2_O_2_ for 20 min. H_2_O_2_ was removed, and after a 10-min additional incubation, hyphae were observed at the indicated times using confocal microscopy. (**A**) Changes in the mitochondria–ER morphology after H_2_O_2_ treatment. (**B**) Magnification of the mitochondria–ER contacts during H_2_O_2_-induced mitochondrial constrictions. The samples were treated as in (**A**), fixed, and observed using Airyscan microscopy. Labels 1 and 2 point to examples of different types of mitochondrial–ER interactions. The red and green signals correspond to mitochondria labelled with mCherry and ER labelled with GFP, respectively. The arrowheads point to mitochondria–ER contacts. Scale bar = 2 μm.

**Figure 6:**
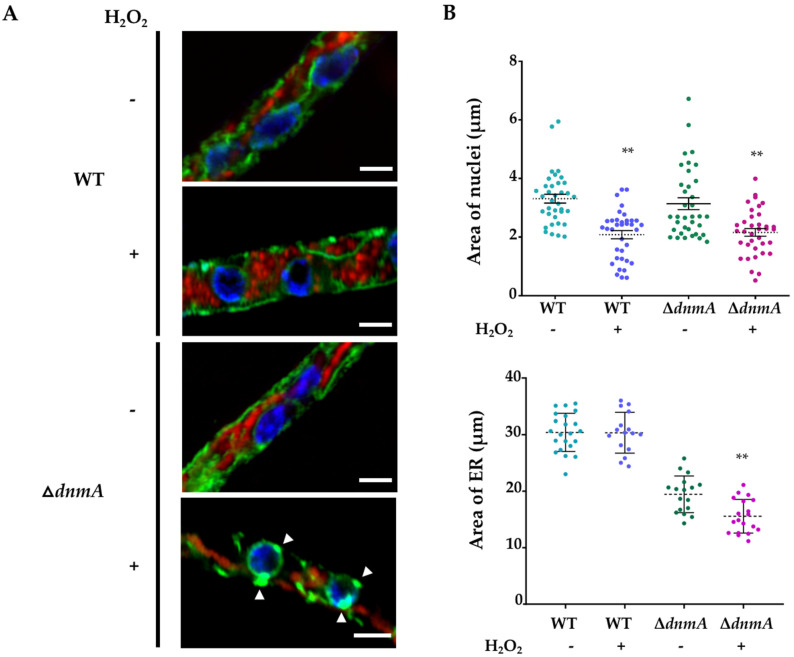
H_2_O_2_ induces morphological changes in mitochondria, ER and nuclei. (**A**) Mycelia from WT (CVG46) and *ΔdnmA* (CVG47) strains grown for 18 h were treated or not with 5 mM H_2_O_2_ for 20 min. H_2_O_2_ was removed, and after a 10 min of additional incubation, hyphae were observed using Airyscan microscopy. (**B**) The area of 35 nuclei from WT and *ΔdnmA* strains treated or not with H_2_O_2_ was determined. Under the same conditions, the total ER area from 35 hyphal tips was determined (** *p* < 0.001 by *t*-test). The red, green and blue signals correspond to mitochondria labelled with mCherry, ER labelled with GFP, and nuclei labelled with BFP, respectively. The white arrowheads point to ER-dotted structures around nuclei. Scale bar = 2 μm.

Previous studies have established that close contact between mitochondria and the endoplasmic reticulum (ER) is important for mitochondrial division [17,38,39]. In order to examine mitochondria–ER interactions during H_2_O_2_-induced mitochondrial constrictions, we generated *ΔdnmA* strain CVG32, in which the mitochondria and ER were labelled with mCherry and GFP, respectively. The in vivo H_2_O_2_ experiments in Figure 5A show that before the constrictions were evident (0 s), the ER appeared as a filamentous network around the mitochondrial filament, with some ER regions adjacent to or in contact with the mitochondrial filament. Between 60 and 120 s, mitochondrial constrictions became evident, and the ER appeared surrounding some of these constrictions and in closer contact with mitochondria. Between 300 and 360 s, the mitochondria’s filamentous shape started to recover, and mitochondria–ER interactions became less evident. In order to better appreciate these interactions in the absence of organelle movement, we decided to make observations in mycelia fixed immediately after the H_2_O_2_ treatment. The results in Figure 5B show several instances in which the ER was clearly surrounding or interacting with mitochondrial constrictions. Under these conditions, at least two different types of mitochondria–ER interactions could be identified: 1, cases in which the ER was encompassing a mitochondrial constriction and 2, cases in which the ER clearly co-localized with the globular structures produced by the constriction of mitochondria. In addition to these changes, we appreciated that the structure of the ER was modified by the H_2_O_2_ treatment. As seen in Figure 5A, the ER network developed dotted structures between 60 and 360 s, which were more evident around putative round nuclear structures. In order to confirm this, we repeated the experiment using a strain in which the nuclei were labelled with BFP. The results in Figure 6A show that in the wild-type strain, H_2_O_2_ induced the formation of a few ER-dotted structures. However, these were not increased around nuclei. In contrast, and as observed before in the *ΔdnmA* mutant (Figure 5A), H_2_O_2_ led to an increased ER signal around nuclei and the production of ER-dotted structures, which were more evident in the nuclear boundary. Additionally, in both strains, H_2_O_2_ induced clear changes in nuclear morphology, from an elongated to a rounded shape. When the fluorescence signal intensity was used to calculate the total ER and nuclear area, the ER area was lower in the *ΔdnmA* mutant compared to the WT strain, suggesting that the lack of DnmA and the resulting tubular mitochondrial morphology seem to affect the shape and extension of the ER network. The presence of H_2_O_2_ further decreased the ER area in the *ΔdnmA* mutant, while H_2_O_2_ induced a reduction in the nuclear area in both the WT and *ΔdnmA* strains (Figure 6B).

In summary, our results show that the ER maintains close contacts with mitochondria even in the absence of division, and that in multiple cases the ER is localized around the mitochondrial constrictions induced by H_2_O_2_, suggesting that mitochondria–ER contacts are necessary in order to produce this H_2_O_2_-induced response. Moreover, we show that H_2_O_2_ induces drastic changes in nuclear structure and, in the absence of mitochondrial division, it induces the formation of ER dot-like structures, particularly around the nuclei.

### 3.3. Mitochondrial Constrictions and ER-Dotted Structures Are Formed during Asexual Development

**Figure 7:**
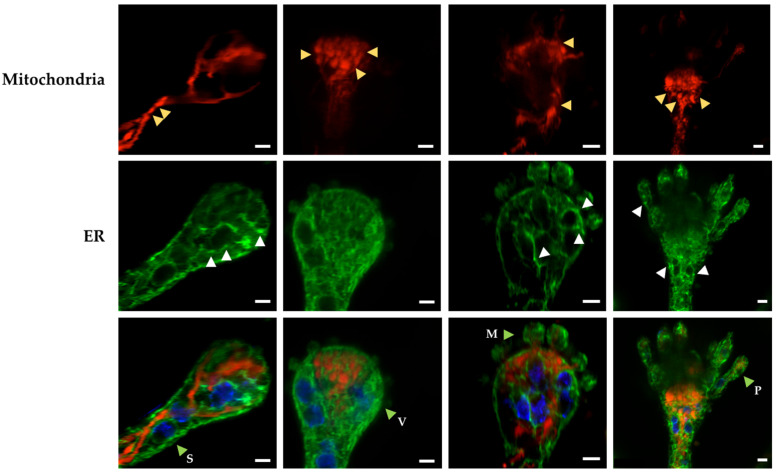
Mitochondrial constrictions and ER-dotted structures are produced during normal conidiophore development. The growing edge of an *ΔdnmA* (CVG47) colony grown for 3 days was sectioned, and its conidiophores were observed using Airyscan microscopy. The red, green and blue signals correspond to mitochondria labelled with mCherry, ER labelled with GFP, and nuclei labelled with BFP, respectively. The yellow arrowheads point to constricted mitochondria; white arrowheads indicate ER-dotted structures. The green arrowheads point to conidiophore stalks (S), vesicles (V), metulae (M) and phialides (P). Scale Bar = 2 μm.

We have proposed that cell differentiation is a response to increased cellular ROS levels [1,2]. Consistent with this idea, we observed that an extensive mitochondrial division is produced not only in response to external H_2_O_2_, but also during conidiophore development. Moreover, in the absence of mitochondrial division, an extensive branching of mitochondria is produced during this differentiation process, allowing the inheritance of a single mitochondrion to the asexual spores [22]. In order to determine whether mitochondrial and ER morphological changes similar to those induced by H_2_O_2_ were detected under physiological conditions, we analyzed the morphology of these organelles at early stages of conidiophore development. The results in Figure 7 show the presence of mitochondrial constrictions at the conidiophore stalk and vesicle developmental stages. Under these conditions, the ER formed an intricate network, parts of which surrounded the nuclei or were found interacting with mitochondria at both the filaments and constriction points. ER dot-like structures were also observed in some cases around the nuclei, which in general presented rounded, rather than elongated, shapes.

These results show that mitochondria, ER and nuclei undergo morphological changes during asexual development that are similar to those induced by H_2_O_2_ during hyphal growth. This suggests that H_2_O_2_ produced under physiological conditions during normal growth and conidiophore development might impact organelle function—including the regulation of mitochondrial constriction and division—at mitochondria–ER interaction points.

### 3.4. Calcium Is Required for Mitochondrial Constrictions Induced by H_2_O_2_ or CCCP but Not for the Remodeling of the Mitochondrial Outer Membrane

**Figure 8:**
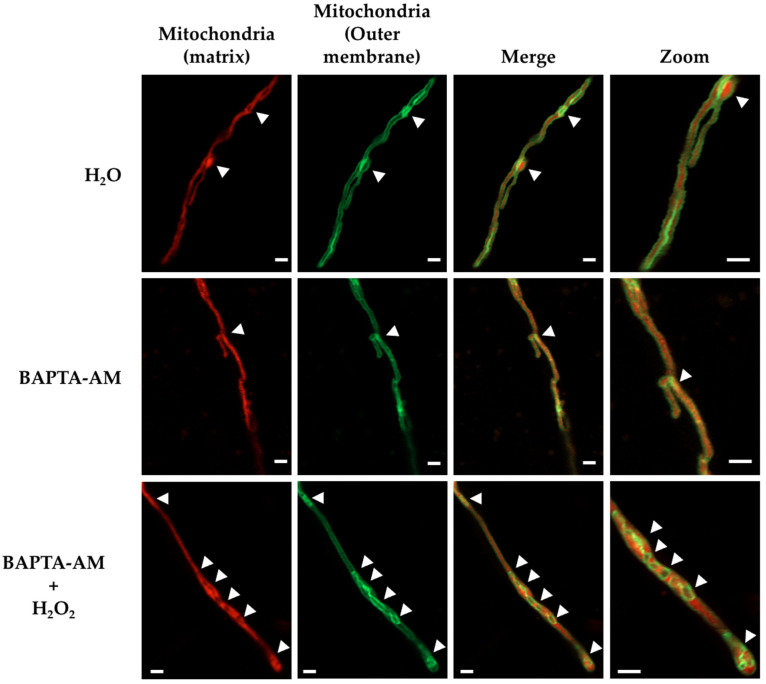
BAPTA-AM prevents mitochondrial constriction induced by H_2_O_2_ but not the remodeling of the mitochondrial outer membrane. Mycelia from the *ΔdnmA* (CVG45) strain were grown for 18 h, incubated with 200 µM BAPTA-AM for 2 h, rinsed with sterile water, treated or not with 5 mM H_2_O_2_ for 20 min, and then observed using Airyscan microscopy. The arrowheads indicate regions where the mitochondrial outer membrane is condensed or forms circular structures. Scale bar = 5 μm.

Mitochondria and the ER make intimate contacts that are necessary for mitochondrial dynamics and in order to exchange calcium (Ca^2+^) and lipids [40]. In order to explore whether Ca^2+^ was necessary for H_2_O_2_-induced mitochondrial constriction, we used BAPTA-AM, a cell-permeant, highly specific Ca^2+^chelator. *ΔdnmA* mutant cells were incubated with BAPTA-AM for 2 h and then treated or not with H_2_O_2_ or CCCP for 20 min, and were observed using confocal microscopy. The incubation with BAPTA-AM alone did not notably affect the tubular morphology of the mitochondria (Figure S6A). Remarkably, the preincubation with BAPTA-AM prevented the formation of mitochondrial constrictions in response to both H_2_O_2_ and CCCP (Figures S6B and S7). Because H_2_O_2_ also induced major rearrangements of the mitochondrial external membrane (Figure 4 and Figure S4), we asked whether BAPTA-AM was also able to prevent these changes. The results in Figure 8 show that *ΔdnmA* tubular mitochondria usually contain a few regions where the mitochondrial outer membrane looks condensed, and the number of these regions was barely affected by the presence of BAPTA-AM. Like before, H_2_O_2_ did not induce mitochondrial constrictions in hyphae pretreated with BAPTA-AM. However, H_2_O_2_ was still able to induce major remodeling of the mitochondrial outer membrane, which was apparent in the formation of circular structures (Figure 8, lower panels). These results indicate that H_2_O_2_ induces mitochondrial constriction and outer membrane rearrangement by two different pathways.

### 3.5. MdmB, a Subunit of the ERMES Complex, Is Required for Mitochondrial Constriction and Division

**Figure 9:**
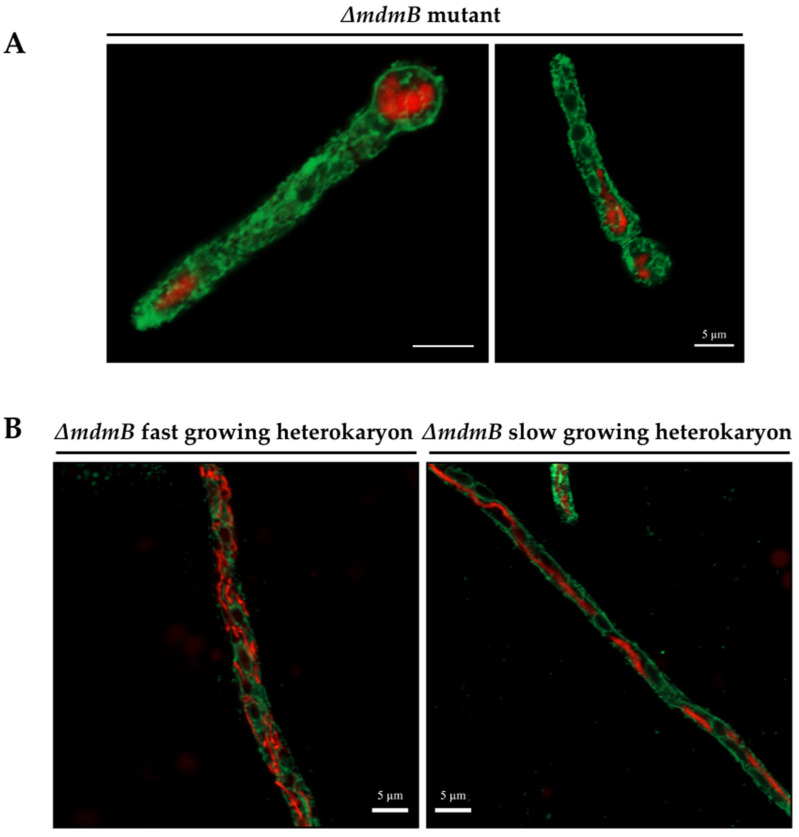
MdmB is required for mitochondrial division. (**A**) Spores from *ΔmdmB* heterokaryon HVG2 were plated on selective media at 37 °C by 21 h. Then, the conidia were fixed and observed using Airyscan microscopy. (**B**) Fast-growing and slow-growing mycelial sectors, obtained from a *ΔmdmB* HVG2 heterokaryotic colony grown on selective media for 5 days, were fixed and observed using confocal microscopy. The red and green signals correspond to mitochondria labelled with mCherry and ER labelled with GFP, respectively. Scale bar = 5 μm.

The *S. cerevisiae* ER–mitochondria encounter structure (ERMES) is a tethering complex composed by proteins Mdm10, Mmm1, Mdm43 and Mdm12; it is critical for ER–mitochondrial interactions [41]. In *A. nidulans*, the protein MdmB (AN6901) corresponds to *S. cerevisiae* Mdm10 [42]. In order to analyze the importance of the ERMES complex and ER–mitochondria interactions in H_2_O_2_-induced mitochondrial constriction and division, we decided to generate *ΔmdmB* mutants using the *AfpyrG* gene as selective marker (Figure S8A). However, after multiple attempts, we were only able to obtain transformants with heterokaryotic morphology (i.e., colonies with irregular growth sectors and heterogenous conidiation), suggesting that *mdmB* was an essential gene in *A. nidulans* (Figure S8B). Indeed, this was confirmed by using PCR to corroborate the heterokaryotic nature of three independent transformants. When conidia from these heterokaryons were plated on selective media, lacking uridine/uracil, the WT conidia failed to germinate, while the *ΔmdmB* conidia were able to germinate and form a short germ tube that failed to grow any further (Figure S8C). This allowed us to determine the terminal phenotype of *ΔmdmB* mutants with respect to mitochondria and ER morphologies. As shown in Figure 9A, *ΔmdmB* mutant germlings contain a few globular mitochondria, instead of the more filamentous mitochondria normally seen in WT strains under these conditions. Notably, these globular mitochondria did not show clear contacts with the ER.

In order to determine the effects of different MdmB dosages on mitochondrial morphology, we compared fast-growing and slow-growing mycelial sectors obtained from *ΔmdmB* heterokaryotic colonies grown on selective media. The results in Figure 9B (left panel) show that the fast-growing sectors contained mitochondria with a wild-type morphology composed of a filamentous and branched network and some smaller individual mitochondria. The ER was observed as a complex network intertwined with the mitochondrial network. In contrast, slow-growing sectors contained some hyphae in which a single mitochondrial filament was observed, similar to those observed in *ΔdnmA* and *ΔfisA* mutants, while the ER formed a simpler and mainly cortical linear network, where mitochondria–ER contacts were not as evident.

**Figure 10:**
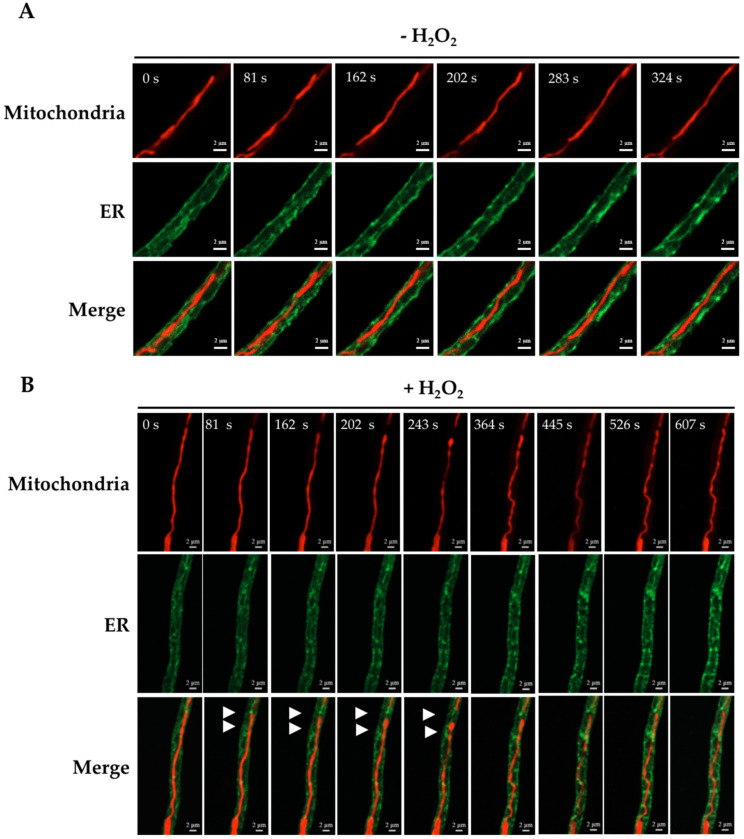
Mitochondria–ER contacts are essential in order to produce mitochondrial constrictions in response to H_2_O_2_. Mycelia from a slow-growing section from *ΔmdmB* heterokaryon HGV2, grown on selective media for 5 days, was treated (**B**) or not (**A**) with 5 mM H_2_O_2_ for 30 min, and was then observed for the indicated times using confocal microscopy. The white arrowheads in (**B**) point to a ER–mitochondria contact point where an incipient mitochondrial constriction is observed.

Using the same approach, we asked whether MdmB was necessary to induce the mitochondrial constrictions elicited by H_2_O_2_. In vivo experiments using hyphae from *ΔmdmB* slow-growing heterokaryotic sectors with tubular mitochondria (Figure 10A) showed that the mitochondria and the ER filamentous form remained virtually unchanged for the observation period (0–324 s). When these sectors were exposed to H_2_O_2_, the mitochondria preserved most of their filamentous form, and only two incipient constrictions were observed between 202 and 243 s at putative mitochondria–ER contact points (Figure 10B). Similarly, CCCP was also largely ineffective to induce mitochondrial constrictions in tubular mitochondria from *ΔmdmB* slow-growing heterokaryotic sectors (Figure S9). These results reveal that MdmB is necessary for the development of mitochondrial constrictions induced by either H_2_O_2_ or CCCP.

**Figure 11:**
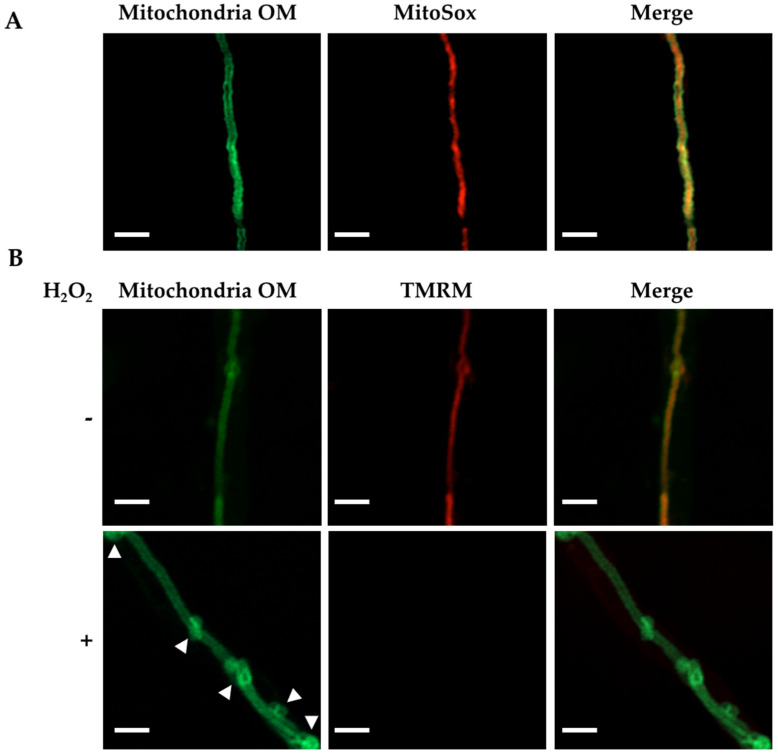
Tubular mitochondria from **Δ*mdmB* heterokaryons show ROS accumulation, and in response to H_2_O_2_ lose membrane potential and undergo the major reshaping of the outer mitochondrial membrane. (**A**) Mycelia from a slow-growing sector from a *ΔmdmB* heterokaryon HVG3 colony grown on selective media for 5 days were stained with MitoSox for 10 min, rinsed with sterile water, and observed using Airyscan microscopy. (**B**) Mycelia obtained as in (**A**) were stained with TMRM for 20 min, rinsed with water, and treated or not with 5 mM H_2_O_2_ for 30 min. The samples were observed using Airyscan microscopy. The white arrowheads indicate rearrangements of the mitochondrial outer membrane (OM). Scale bar = 5 μm.

All of the long mitochondrial filaments observed in *ΔmdmB* hyphae from slow growing heterokaryotic sectors were found to accumulate ROS when stained with the superoxide reporter MitoSox Red (Figure 11A), which is consistent with our previous results showing that the lack of mitochondrial division results in an increased production of mitochondrial ROS [22]. As shown in Figure 11B, *ΔmdmB* heterokaryon tubular mitochondria were stained with TMRM and depolarized by the addition of H_2_O_2_, indicating that mitochondria–ER contacts mediated by the ERMES complex are needed for H_2_O_2_-induced mitochondrial constriction but not for initial H_2_O_2_-induced mitochondrial membrane depolarization. Notably, although mitochondrial constrictions were not observed under these conditions, H_2_O_2_ was still able to induce the major reshaping of the outer mitochondrial membrane.

In summary, our results indicate that *A. nidulans* ERMES complex component MdmB is an essential protein that mediates contacts between mitochondria and the ER, and that these contacts are necessary for H_2_O_2_-induced mitochondrial early constriction and later division but not for the rearrangement of the outer mitochondrial membrane. The fact that Ca^2+^ is necessary for H_2_O_2_-induced mitochondrial constriction but not for the remodeling of the outer mitochondrial membrane (Figure 8) supports a model in which H_2_O_2_ regulates the transport of Ca^2+^ between the ER and the mitochondria to regulate mitochondrial constriction and division.

## 4. Discussion

### 4.1. H_2_O_2_ Induces Mitochondrial Depolarization, Constriction and Rearrangements of the Outer Mitochondrial Membrane 

H_2_O_2_ and CCCP induce extensive mitochondrial division in *A*. *nidulans* and other eukaryotes. However, earlier events occurring before actual mitochondrial division are difficult to appreciate in a wild-type background. Using mutants impaired in mitochondrial division, we found that these two compounds induce a profuse production of mitochondrial constrictions as well as the reshaping of the outer mitochondrial membrane. CCCP induced rapid depolarization and a generalized formation of mitochondrial constrictions in a process that was irreversible. H_2_O_2_ induced gradual depolarization followed by widespread formation of mitochondrial constrictions in a process that was reversible within a few minutes. In addition, the formation of isolated, spontaneous and reversible constrictions was observed in the course of normal growth. These results suggest that the mitochondrial constrictions which are normally present during regular growth result from local depolarization events, the frequency of which is markedly increased by H_2_O_2_. In any case, the dramatic changes in mitochondrial morphology induced by external H_2_O_2_ involve the presence of H_2_O_2_ perception mechanisms that should also function under normal conditions.

Lee and Yoon reported the occurrence of transient contractions of the mitochondrial matrix that were notably increased by a lack of mitochondrial division. However, these authors proposed that contractions led to a reversible loss of inner membrane potential [43]. We did not observe that the lack of mitochondrial division per se resulted in an extensive production of mitochondrial constrictions. Moreover, in the presence of both H_2_O_2_ and CCCP, the loss of inner membrane potential preceded the massive production of mitochondrial constrictions. Our results indicate that in the absence of H_2_O_2_, contractions are not generalized, despite the apparent continuity of the mitochondrial filaments present when DnmA, FisA or MdmB are absent. This might be related to the fact that individual cristae within the same mitochondrion have different membrane potentials and are functionally independent [44]. More recently, Cho et al. reported that the spontaneous and repetitive constriction of the mitochondrial inner compartment (CoMIC) was associated with a subsequent division in neurons, supporting the idea that CoMICs are priming events for efficient mitochondrial division. However, in their experiments, CoMICs were observed in the internal mitochondrial membrane independently of outer mitochondrial morphological changes [45]. We generally observed that H_2_O_2_-induced mitochondrial constrictions involved both internal and outer mitochondrial membranes. However, H_2_O_2_ induced additional changes in the outer mitochondrial membrane that remained after the mitochondrial tubular shape was restored, which resulted in the formation septum and donut-like structures, and in some cases led to mitochondrial branching.

In addition to its effects on mitochondrial morphology, H_2_O_2_ induced the formation of ER dot-like structures and the rounding of nuclei. Notably, similar changes were observed in conidiophore vesicles for asexual sporulation. In a wild-type background, mitochondria undergo extensive division at the vesicle stage [22]. In the absence of mitochondrial division, the observed extensive mitochondrial constrictions and branching are necessary to distribute mitochondria to the multiple metulae that differentiate from the vesicle, and from there to developing phialides and conidial cell types ([22] and this work). One possible interpretation is that H_2_O_2_ production increases during conidiophore development as a mechanism to regulate mitochondrial division and inheritance. Consistent with this is the fact that mutants lacking ROS-producing NADPH oxidase NoxA and its regulatory subunit NoxR are not only blocked in sexual development [6,46] but also show decreased asexual sporulation [46].

### 4.2. Intracellular Ca^2+^ and MdmB, a Subunit of the ERMES Complex, Are Required for H_2_O_2_-Induced Mitochondrial Constriction and Division

Our results show that mitochondrial depolarization by CCCP or H_2_O_2_ is not enough to induce mitochondrial constrictions, and that intracellular calcium is essential for this process. This is consistent with the fact that, in animal cells, CoMICs occurred at potential division sites contacting the ER, and the intra-mitochondrial influx of Ca^2+^ induced and potentiated CoMIC formation [45].

Indeed, we observed that the ER is in close contact with mitochondria even in the absence of division, and that in many cases the ER is localized around the mitochondrial constrictions induced by H_2_O_2_. Moreover, we showed that MdmB, an ortholog of *S. cerevisiae* ERMES complex subunit Mdm10, is involved in maintaining contact between the ER and mitochondria, and is critical for H_2_O_2_-induced mitochondrial constriction and division. The elimination of components of the ERMES complex has different consequences in different fungi, but generally mitochondrial morphology is affected. In *A. nidulans*, MdmB was found to be an essential protein. Previously, *A. nidulans* mutants with a partially deleted *mdmB* gene were reported as viable, although with reduced growth at 37 °C. However, at a low temperature these mutants contained both tubular and giant non-motile mitochondria [42], suggesting that these mutants expressed a MdmB polypeptide with partial function. The characterization of *A. fumigatus* conditional mutants in all four core ERMES components and *mdm10* and *mdm12* deletion mutants showed that each component was critical for growth, and that the downregulation of each component produced cells with giant as well as very small mitochondria [47]. Similarly, in our experiments, the terminal phenotype of *ΔmdmB* mutants was characterized for the formation of ball-like bloated mitochondria. The downregulation of MdmB in slow-growing heterokaryons resulted in a novel phenotype characterized by a lack of mitochondrial division and the production of tubular mitochondria with increased ROS levels, exactly as occurs in non-dividing mitochondria from *ΔdnmA* and *ΔfisA* mutants. A defective mitochondrial division could explain the defects in mitochondrial inheritance observed in *S. cerevisiae mdm10* mutants [48].

### 4.3. H_2_O_2_ Differential Roles in Mitochondrial Dynamics

Our results show that H_2_O_2_ affects mitochondrial function in at least three different aspects. It induces gradual membrane depolarization, mitochondrial constriction and outer membrane remodeling. Although interrelated, these processes can be separated, as shown by the fact that Ca^2+^ and a close contact between mitochondria and ER are necessary for mitochondrial constriction but not for mitochondrial depolarization or the reshaping of the outer membrane. ROS have also been reported as affecting mitochondrial distribution and mobility, but these effects were independent of cytoplasmic Ca^2+^ or the mitochondrial membrane potential [49].

The relationship between mitochondrial membrane depolarization, ROS and Ca^2+^ is a complex subject [50,51]. For instance, CCCP-induced mitochondrial depolarization led to an increased oxidation of nuclear DNA in animal cells, suggesting an increased production of ROS under these conditions [51]. On the other hand, there is evidence indicating that transient depolarization can lead to the suppression of ROS production [52]. In addition, non-dividing mitochondria, either because they lack DnmA, FisA [22] or MdmB (this work), contain increased levels of superoxide and perhaps other ROS, which seem to show a heterogenous concentration within mitochondria.

Despite this, it is known that the low-conductance mitochondrial permeability transition pore regulates the flux of Ca^2+^ and ROS out of the mitochondrial matrix, whilst the accumulation of Ca^2+^ in the mitochondrial matrix induces the opening of a high-conductance mitochondrial permeability transition pore. While mitochondria experience brief depolarization events or flickerings that contribute to the generation of ROS, the opening of the high conductance permeability transition pore leads to complete and persistent mitochondrial depolarization and the redistribution of ions and solutes up to 1.5 kDa across the internal mitochondrial membrane, and a more generalized production of ROS [53]. Although not in connection to mitochondrial constriction and membrane remodeling, a recent publication relates mitochondrial depolarization, ROS and Ca^2+^ signaling between mitochondria and the ER. Boot et al. (2021) reported that transient depolarizations of individual mitochondria, initiated by openings of the permeability transition pore, generate oxidative bursts at the ER–mitochondrial interface. Such oxidative bursts constitute a mitochondria–ER signaling pathway by sensitizing ER redox-sensitive IP3R Ca^2+^ release channels. These authors also proposed that metabolic stress could amplify local ER–mitochondria proximity signals to larger regions of the cell [54].

According to this, our results are consistent with a model in which H_2_O_2_ would increase the frequency of flickering, leading to a gradual mitochondrial depolarization and an increased efflux of endogenous ROS. ROS produced by mitochondrial flickering would sensitize ER redox-sensitive Ca^2+^ release channels, inducing mitochondrial Ca^2+^ loading by the ER, eventually leading to the induction of high-conductance mitochondrial permeability transition in a reversible way, such that the mitochondrial constrictions are reversible. These initial events would be necessary but not sufficient to induce extensive mitochondrial constriction. This interplay between ROS and ER-derived Ca^2+^ can regulate mitochondrial constrictions by affecting actin polymerization. In animal cells, mitochondrial depolarization induces a rapid actin assembly around mitochondria by two parallel pathways, one of them involving cytoplasmic Ca^2+^, PKCB, Rac, WRC and the Arp2/3 complex, which affects mitochondrial morphology by inhibiting mitochondrial circularization and the processing of OPA1 [55]. A similar mechanism, initiated by H_2_O_2_, could result in the mitochondrial constrictions that we observe in *A. nidulans*.

We also found that H_2_O_2_ regulates mitochondrial outer membrane remodeling by a mechanism that is independent of Ca^2+^ and mitochondrial–ER contacts. It is likely that such membrane reshaping results from the mitochondrial membrane fusion events that normally precede mitochondrial division [56]. It is known that different types of stress activate mitochondrial fusion in animal cells in a process regulated by mitofusins Mfn1 and Mfn2, located in the mitochondrial outer membrane, and by Opa1, located in the inner mitochondrial membrane. Notably, oxidized glutathione disulfide promotes the assembly of higher-order Mfn complexes, mediated by reversible disulfide bonds [57,58]. However, Fzo1 proteins, the functional equivalents of animal Mfn in fungi, lack the two conserved cysteines that are essential for Mfn-redox regulation, which led to the suggestion that stress-induced mitochondrial fusion is a specific adaptation to multicellular life [57]. However, our results suggest that alternative mechanisms for a redox regulation of mitochondrial outer membrane fusion are present in fungi. Therefore, although the necessity of mitochondrial constriction for proper Drp1/DnmA oligomerization around mitochondria and their subsequent division seems conserved in fungi and animals, the mechanisms triggering and accomplishing mitochondrial constriction might be different. Here, it is interesting to note that similarly to bacterial division, and in contrast to animal cells, mitochondrial constriction in the red algae *Cyanidioschyzon merolae* is mediated by the protein FtsZ [19,59]. In any case, our results indicate that H_2_O_2_ is a critical signal that regulates mitochondrial dynamics by regulating mitochondrial membrane depolarization, constriction, and outer membrane remodeling.

## Data Availability

Not applicable.

## Supplementary Materials

The following supporting information can be downloaded at: https://www.mdpi.com/article/10.3390/jof8080829/s1. Table S1: *Aspergillus nidulans* strains used in this work; Table S2: Primers used in this work; Figure S1: H_2_O_2_ and CCCP induce mitochondrial division in *A. nidulans*; Figure S2: Number of hyphae containing filamentous or constricted mitochondria in *Δ**dnmA* and *Δ**fisA* mutants; Figure S3: Number of hyphae showing TMRM-stained mitocondria in *Δ**dnmA* mutants treated with H_2_O_2_; Figure S4: H_2_O_2_ induces transient mitochondrial constrictions and the irreversible reshaping of the mitochondrial outer membrane, including the formation of donut-like and septum-like structures; Figure S5: CCCP induces irreversible mitochondrial constrictions and the major reshaping of mitochondrial outer membrane; Figure S6: BAPTA-AM does not affect the mitochondrial filamentous form but prevents mitochondrial constriction induced by H_2_O_2_ or CCCP; Figure S7: BAPTA-AM prevents mitochondrial constriction induced by H_2_O_2_ or CCCP; Figure S8: PCR confirmation of *Δ**mdmB* heterokaryons and the terminal phenotype of *Δ**mdmB* mutants; Figure S9: Filamentous mitochondria from *Δ**mdmB* heterokaryons do not form constrictions in the presence of CCCP.
