# Supplementary material for: H2O2 Induces Calcium and ERMES Complex-Dependent Mitochondrial Constriction and Division as Well as Mitochondrial Outer Membrane Remodeling in Aspergillus nidulans
Source: J Fungi (Basel). 2022 Aug 9;8(8):829. doi: 10.3390/jof8080829 (PMC9410301; doi:10.3390/jof8080829)
Supplement: Supplementary file 1 [file jof-08-00829-s001.zip › jof-1822488-supplementary.pdf]

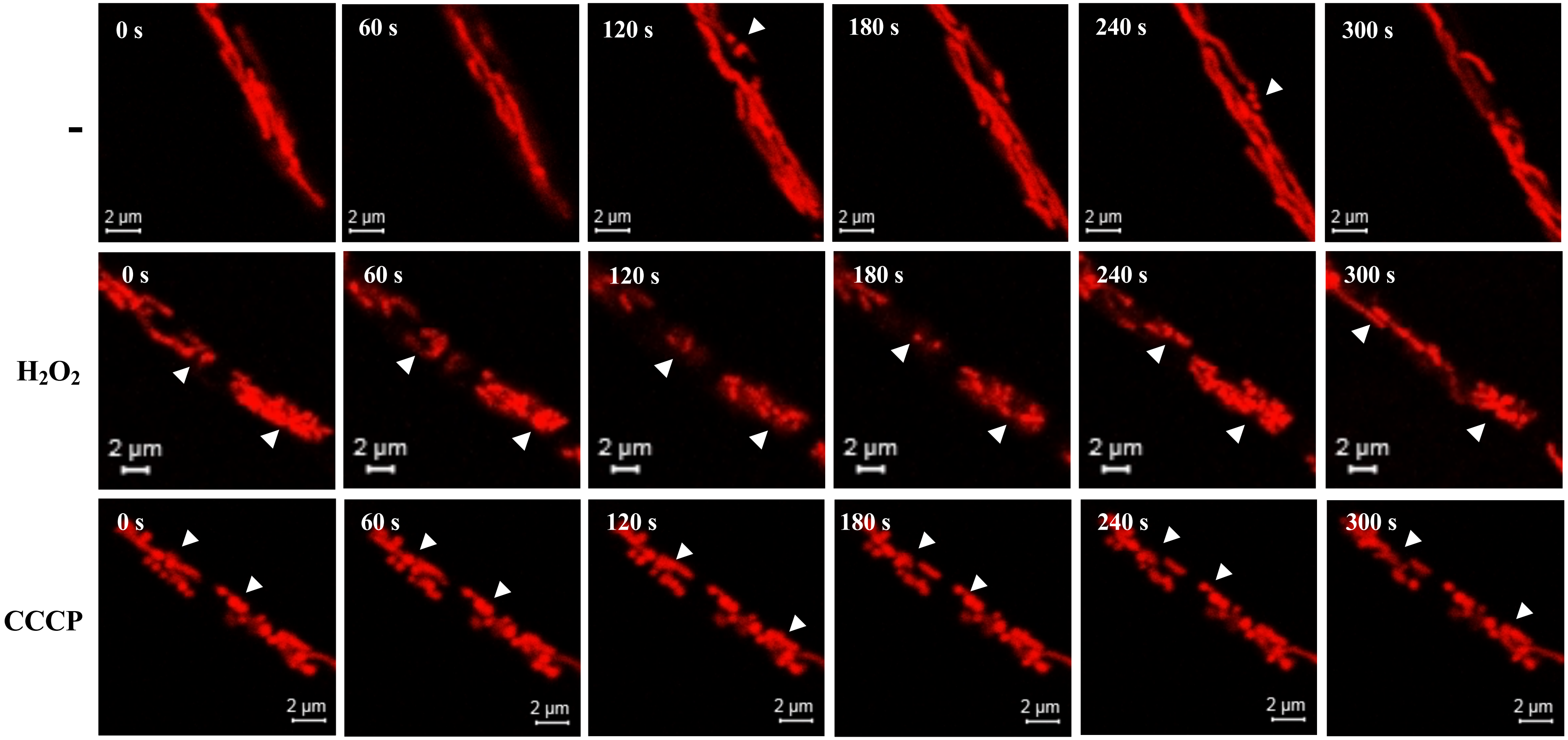

Figure S1. H_2O_2 and CCCP induce mitochondrial division in *A. nidulans*. Mycelia from WT (TRV1) strain grown for 18 h was treated or not with 5 mM H_2O_2 or 10 µM CCCP for 20 min. After this, H_2O_2 or CCCP were removed and after another 10 min incubation, hyphae were observed at the indicated times using confocal microscopy. Mitochondrial matrix is labelled with mts::mCherry. Arrowheads point to some individual or groups of individual mitochondria.

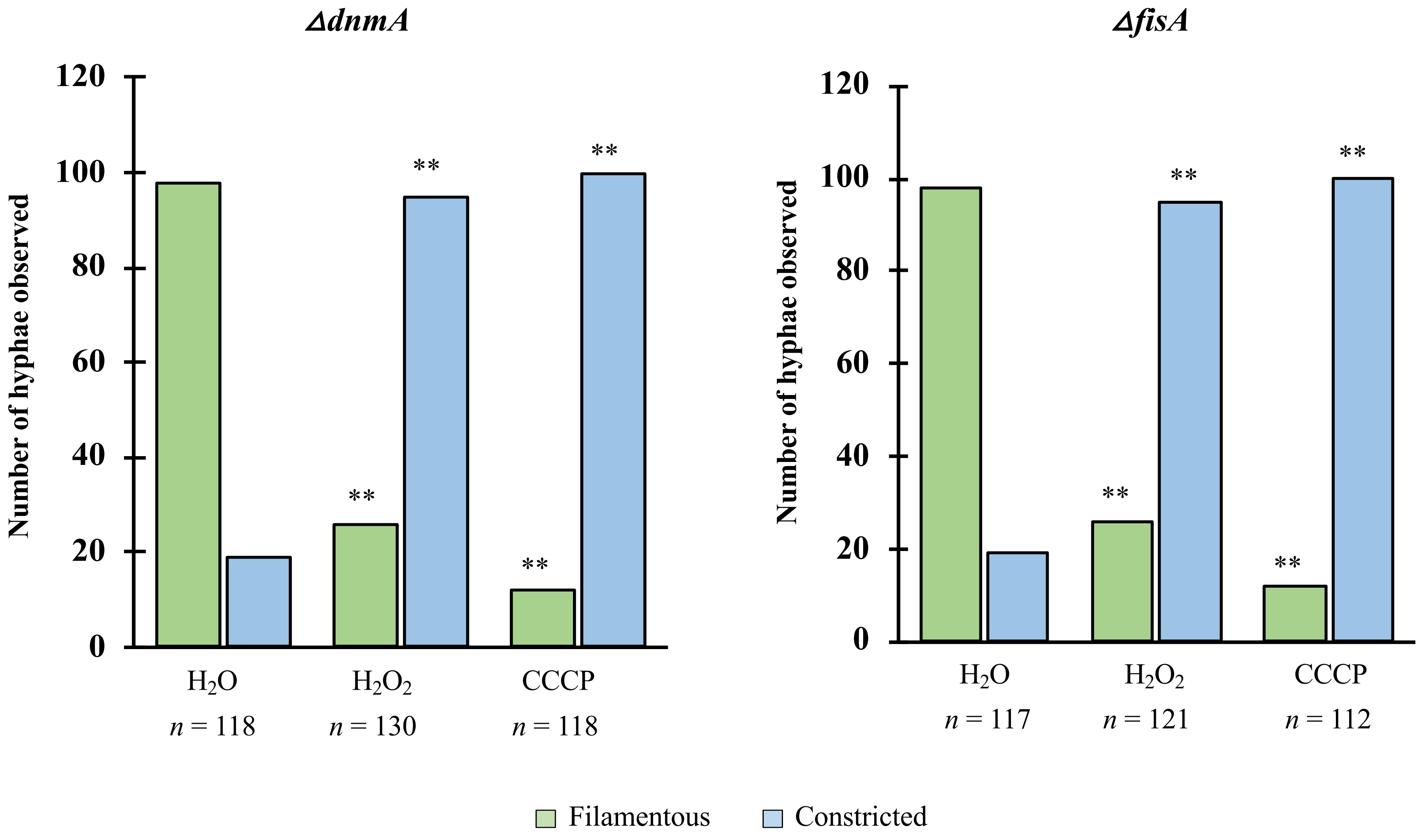

Figure S2. Number of hyphae containing filamentous or constricted mitochondria in *ΔdnmA* and *ΔfisA* mutants. Mycelia from strains CVG1 (*ΔdnmA)* and CVG2 (*ΔfisA*) grown for 18 h was treated or not with 5 mM H_2O_2 or 10 µM of CCCP for 20 min. After H_2O_2 or CCCP were removed, samples were fixed and the number of hyphae showing either filamentous or constricted mitochondria was determined using epifluorescence microscopy (** *P< 0.001* by χ^2- test).

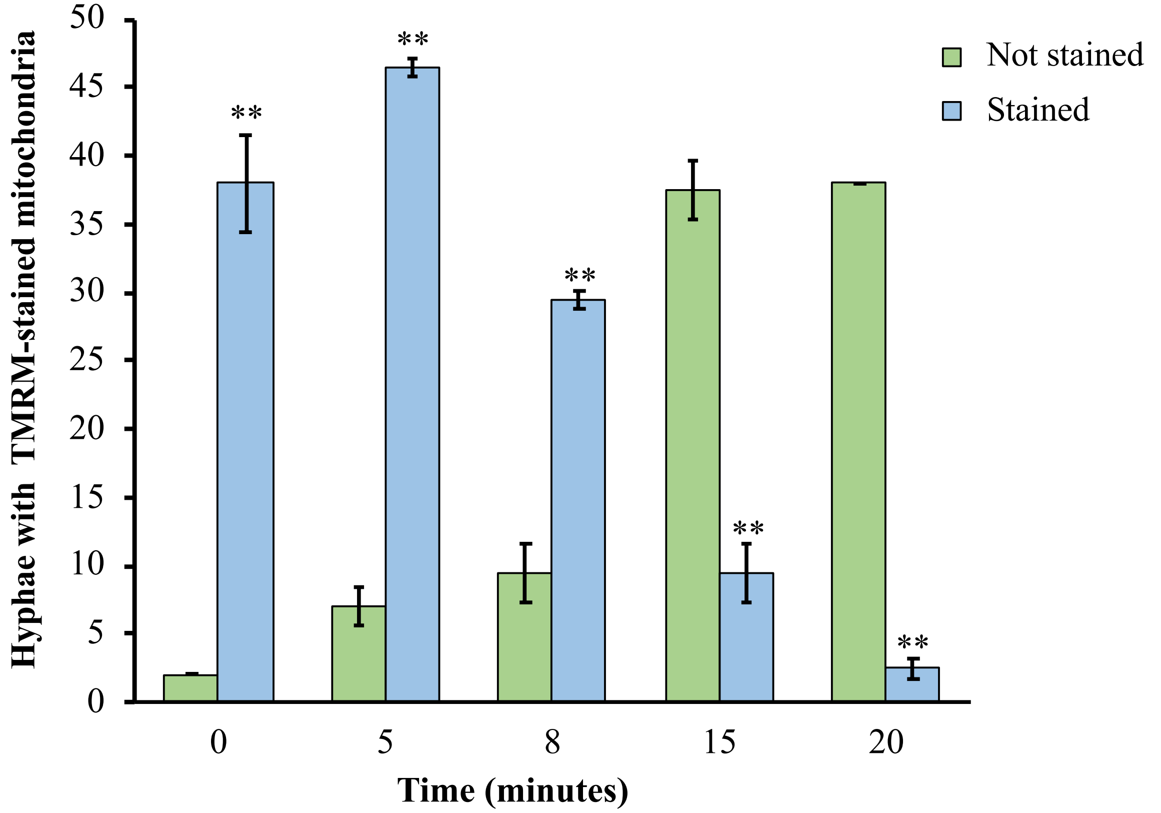

Figure S3. Number of hyphae showing TMRM-stained mitochondria in *ΔdnmA* mutants treated with H_2O_2. Mycelia from *ΔdnmA* strain CVG48 was grown for 18 h at 37 °C, stained with TMRM during 20 min and then treated with 5 mM H_2O_2. At the indicated times, the number of hyphae containing TMRM-stained mitochondria was determined using epifluorescence microscopy (** $P < 0.001$ by *t*- test). $n = 3$ experiments (> 220 hyphae).

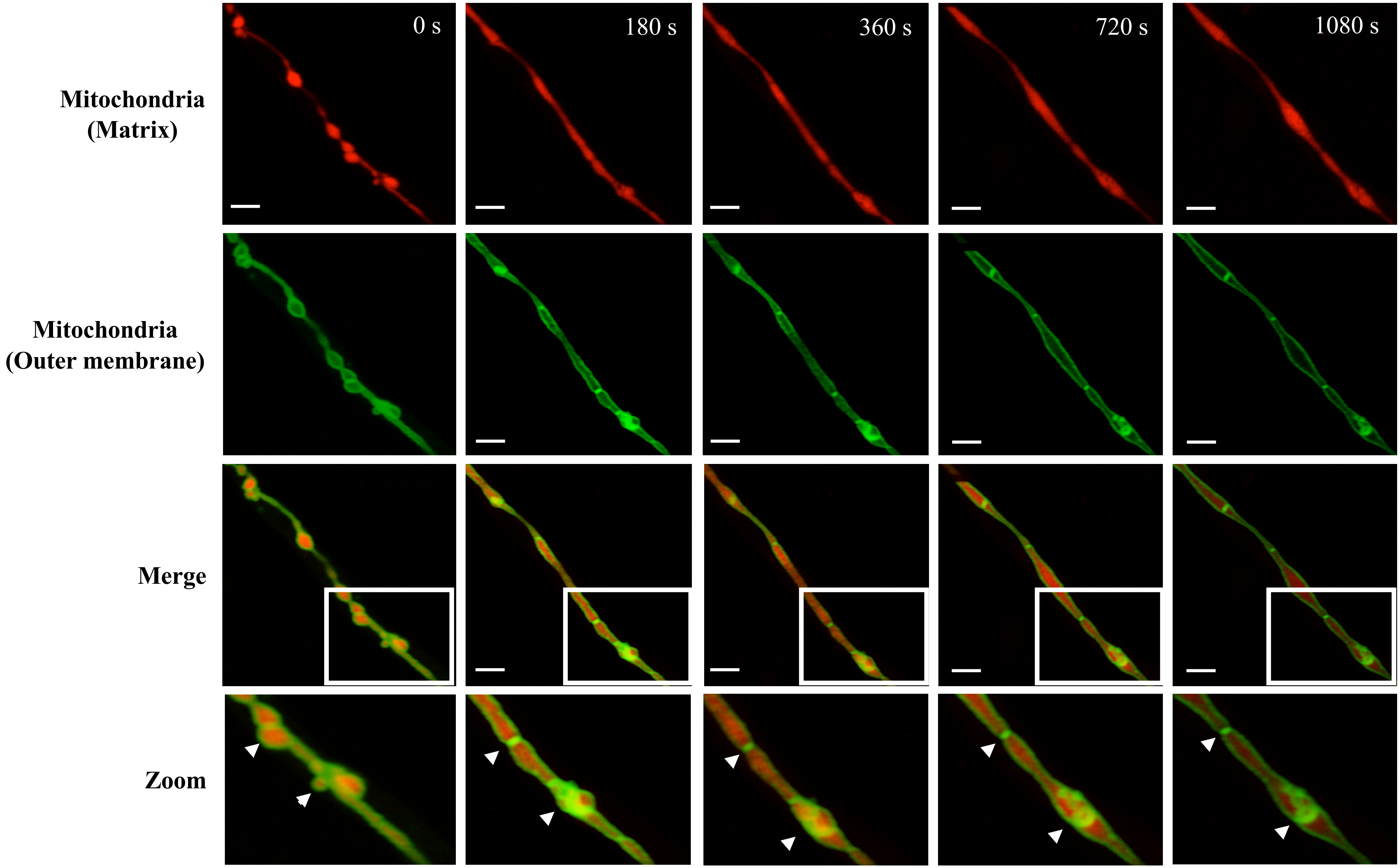

Figure S4. H_2O_2 induces transient mitochondrial constrictions and irreversible reshaping of mitochondrial outer membrane including the formation of donut-like and septum-like structures. Mycelia from *ΔdnmA* mutant strain CVG45 was grown for 18 h and then treated with 5mM H_2O_2 for 20 min. H_2O_2 was removed and after another 10 min incubation, hyphae were observed at the indicated times (seconds) using confocal microscopy. White arrow heads indicate mitochondrial outer membrane major rearrangements, donut-like and septum-like structures. Scale bar = 2μm.

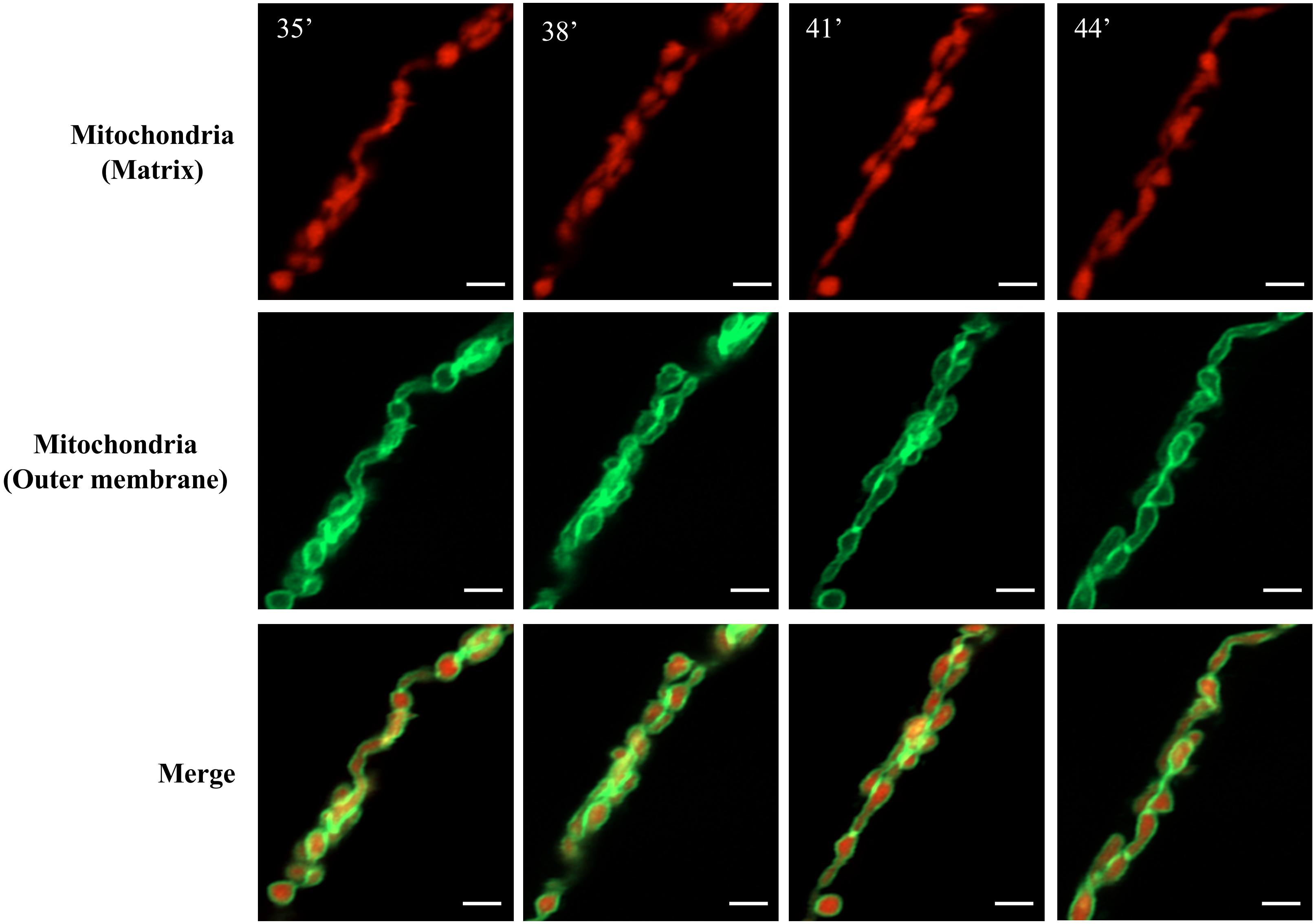

Figure S5. CCCP induces irreversible mitochondrial constrictions and major reshaping of mitochondrial outer membrane. Mycelia from *ΔdnmA* mutant strain CVG45 was grown for 18 h and treated with 10 µM CCCP. CCCP was removed and after another 10 min incubation, hyphae were observed at the indicated times (minutes) using confocal microscopy. Scale bar = 2µm.

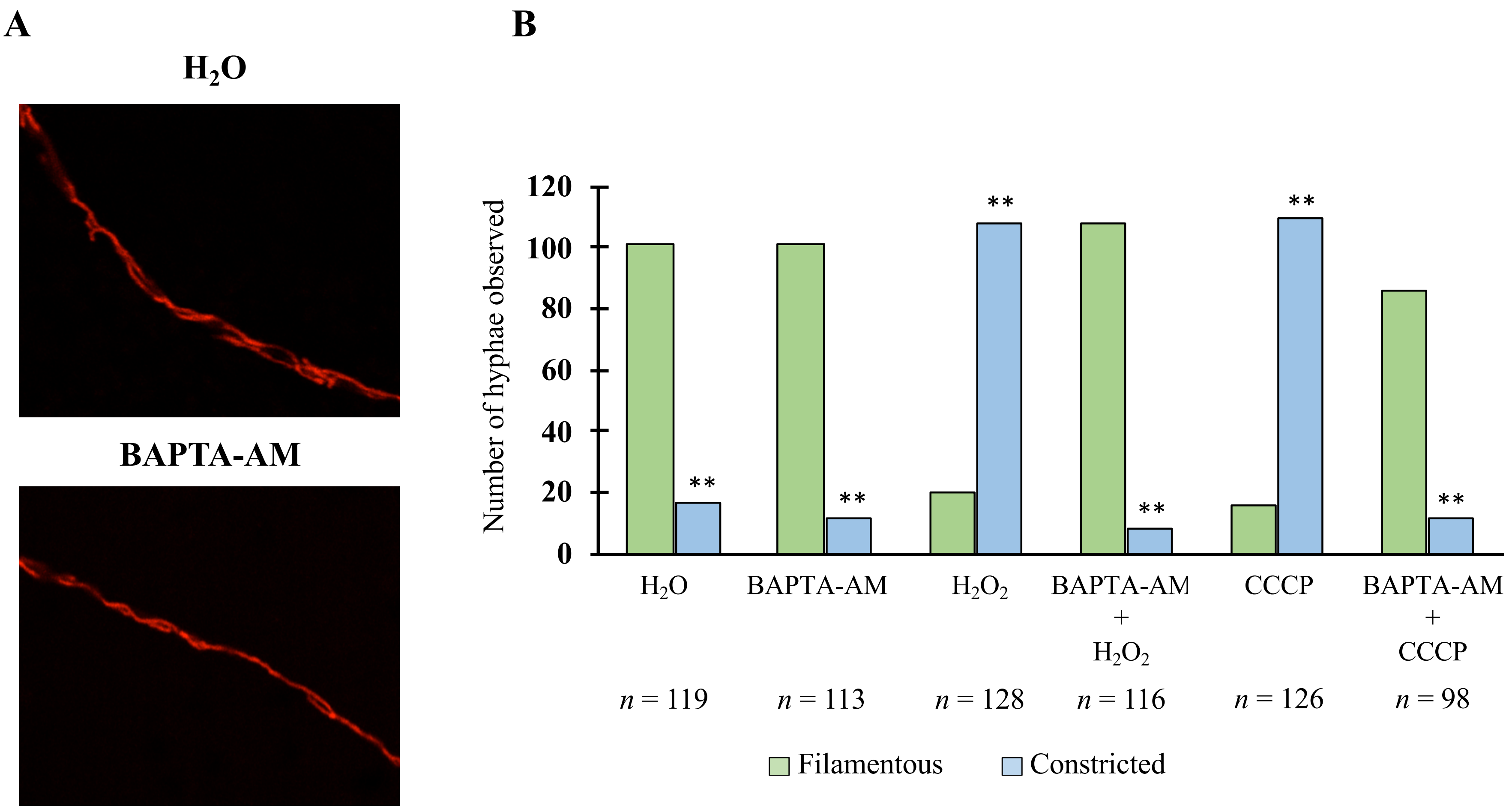

Figure S6. BAPTA-AM does not affect mitochondrial filamentous form but prevents mitochondrial constriction induced by H_2O_2 or CCCP. Mycelia from *ΔdnmA* strain CVG1 was grown for 20 h at 37 °C and incubated with 200 µM BAPTA-AM for 2 h and then treated or not with 5 mM H_2O_2 or 10 µM CCCP during 20 min. After this, samples were washed three times with sterile H_2O and the number of hyphae showing mitochondrial constrictions was determined using epilfluorescence microscopy (** $P < 0.001$ by *t*- test). **A**. Mitochondrial morphology after H_2O or BAPTA-AM treatments. **B**. Number of hyphae showing mitochondrial constrictions after the indicated treatments.

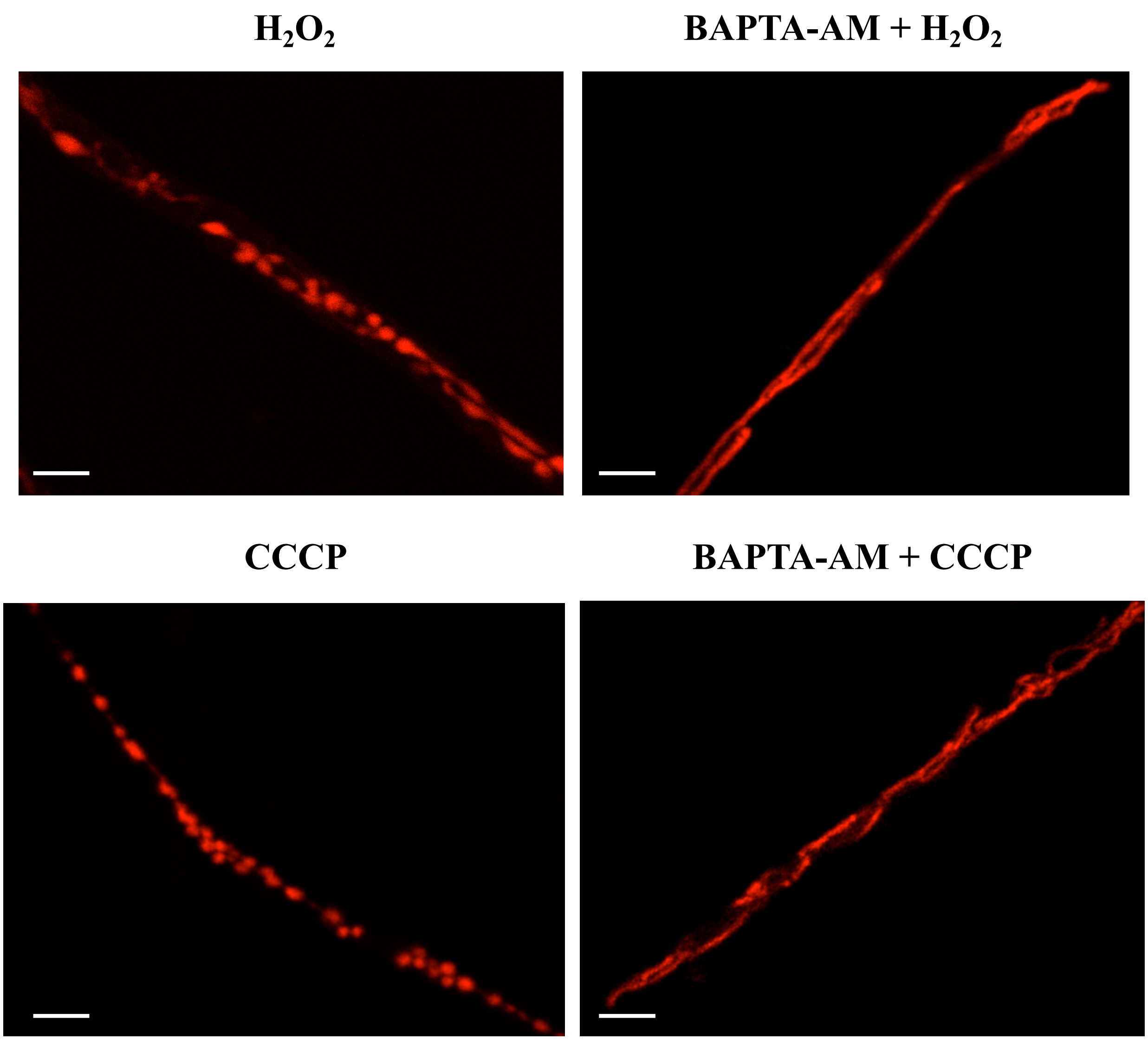

Figure S7. BAPTA-AM prevents mitochondrial constriction induced by H_2O_2 or CCCP. Mycelia from *ΔdnmA (*CVG47) strain was grown for 18 h, incubated with 200 µM BAPTA-AM for 2 h, rinsed with sterile water and then treated or not with 5 mM H_2O_2 or 10 µM CCCP during 20 min and then observed using confocal microscopy Scale bar = 5 µm.

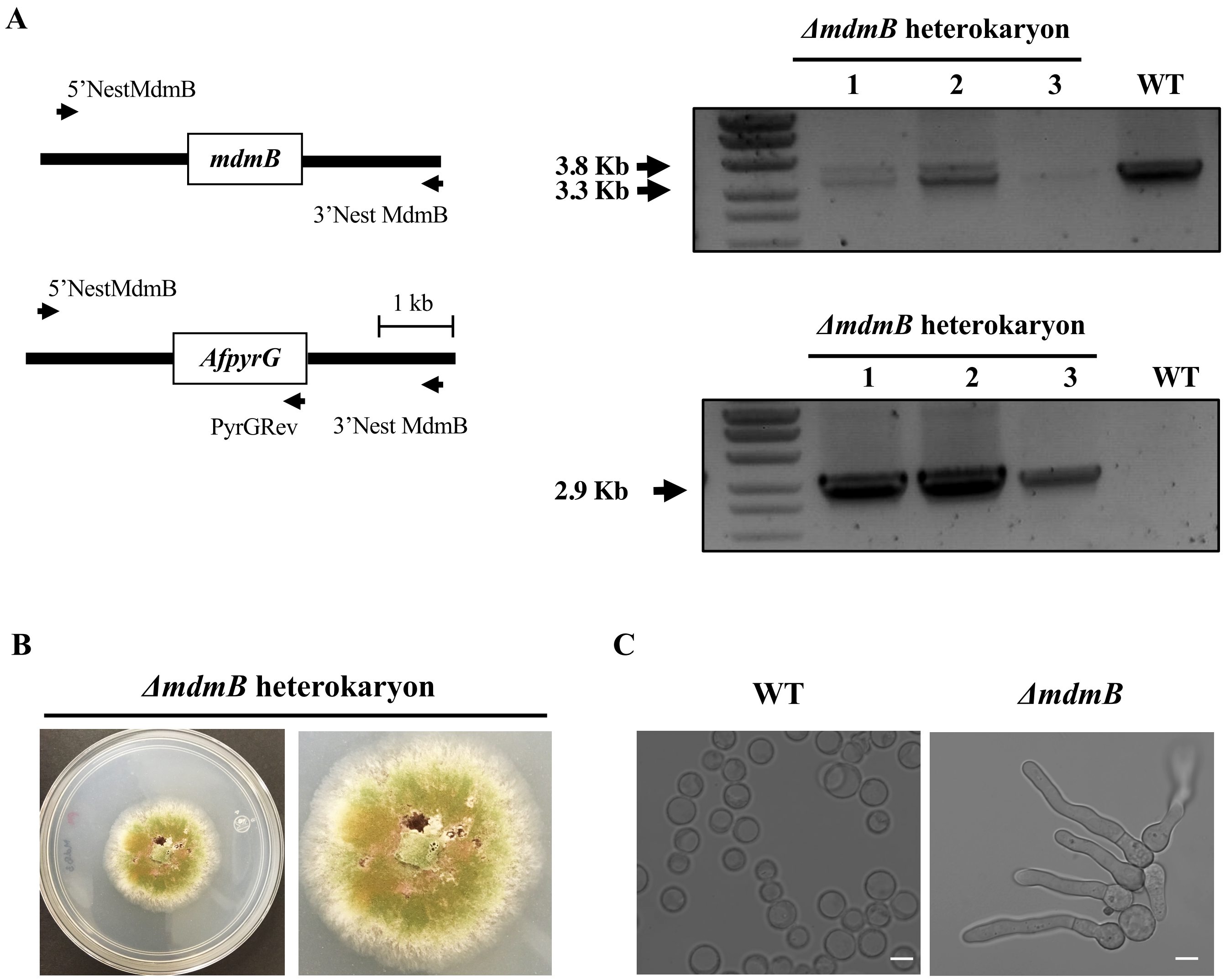

Figure S8. PCR confirmation of *ΔmdmB* heterokaryons and terminal phenotype of *ΔmdmB* mutants. **A.** A *mdmB* deletion construct containing the *AfpyrG* gene, as selective marker, was used to transform strains CVG29 and CDV01. One derived from strain CVG29 (1) and two from strain CDV01 (2 and 3) were confirmed as heterokaryons by PCR using primers 5’NestMdmB and 3’NestMdmB. These primers generated 3.3 and 3.8 Kb products, corresponding to wild type and *ΔmdmB* alleles, respectively. With primers 5´NestMdmB and PryGRev only heterokaryons generated a 2.9 Kb PCR product. **B.** Mycelia from *ΔmdmB* heterokaryon HVG2 grown on supplemented minimal medium for 5 days. **C.** Spores from WT strain CVG29 and *ΔmdmB* heterokaryon HVG2 were plated on selective media during 21 h and then observed using bright field confocal microscopy. Scale bar = 5 µm.

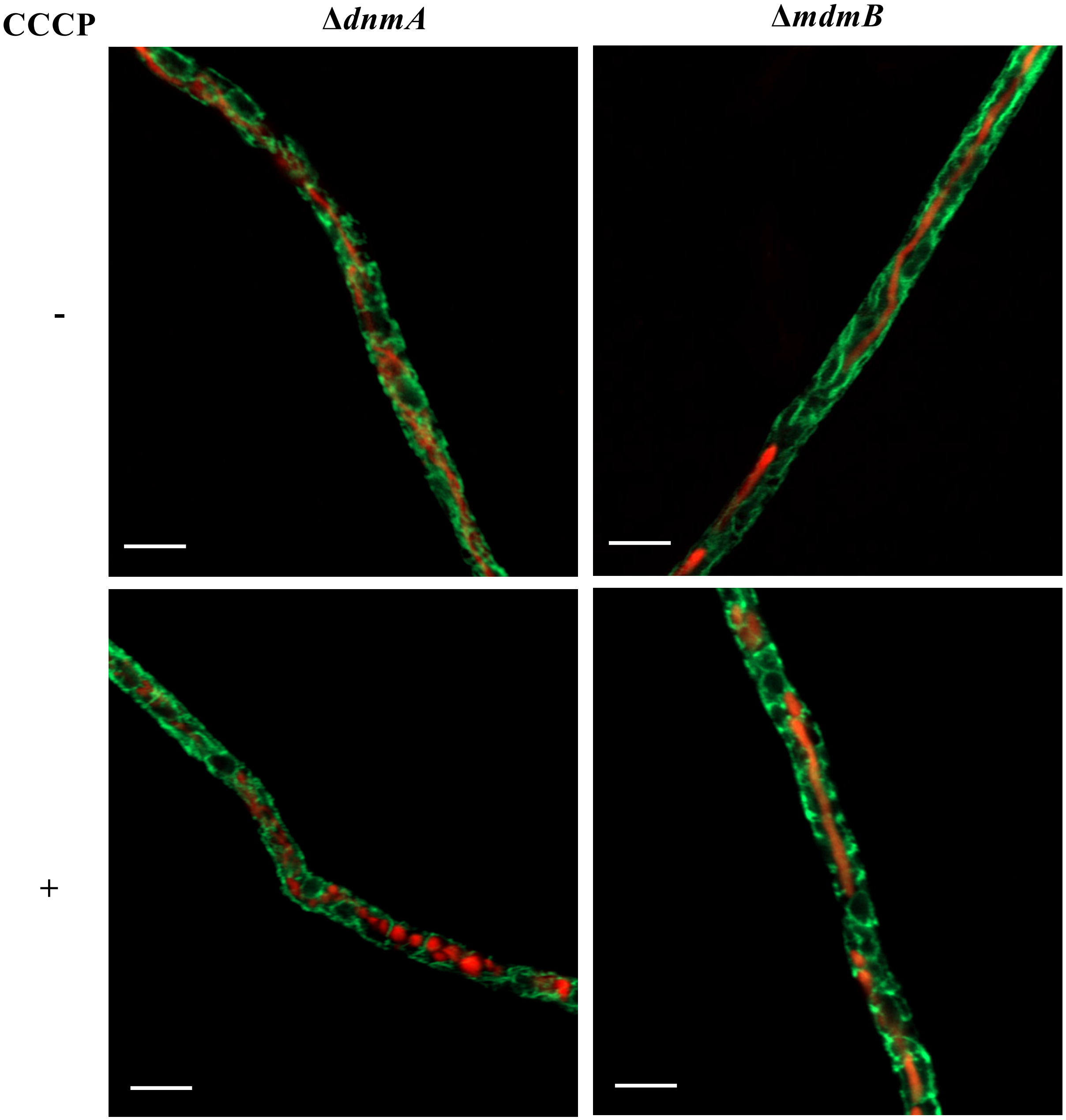

Figure S9. Filamentous mitochondria from *ΔmdmB* heterokaryons do not form constrictions in the presence of CCCP. Slow growing mycelia from *ΔmdmB* heterokaryon HVG2 grown on selective media for 5 days was treated or not with 10 µM CCCP for 20 min. After 10 minutes images were obtained using confocal microscopy. *ΔdnmA* mutant CVG32 (left panels) is shown as control. Red and green signals correspond to mitochondria labelled with mCherry and ER labelled with GFP, respectively. Scale bar = 5 µm.
